# Evidence-Based Medicine Course in Combination With Journal Clubs to Promote Evidence-Based Surgery

**DOI:** 10.7759/cureus.37318

**Published:** 2023-04-09

**Authors:** Dirk T Ubbink, Simone Augustinus, Tim M Feenstra, Nine De Graaf, Stéphanie M Van der Burgt, Mark J Koelemaij, Els J Nieveen van Dijkum

**Affiliations:** 1 Surgery, Amsterdam University Medical Centers, Amsterdam, NLD; 2 Medicine, University of Amsterdam, Amsterdam, NLD; 3 Vascular Surgery, Amsterdam University Medical Centers, Amsterdam, NLD

**Keywords:** phd student, care professionals, staff, surgery, critical appraisal, journal club, evidence-based medicine

## Abstract

Introduction

To provide high-quality surgical care, surgeons must critically appraise medical literature to adapt their clinical practice whenever convincing evidence emerges. This will promote evidence-based surgery (EBS). Over the last decade, we have organized monthly journal clubs (JCs) and more extensive quarterly EBS courses for surgical residents and PhD students, supervised by surgical staff. We evaluated the participation, satisfaction, and knowledge gained by this EBS program, to make the program future-proof and aid other educators.

Materials and methods

An anonymous digital survey was distributed via email among residents, PhD students, and surgeons of the Amsterdam University Medical Centers' (UMC) surgical department in April 2022. The survey included general questions on EBS education, specific course-oriented questions for the residents and PhD students, and questions about supervision for surgeons.

Results

The survey was completed by 47 respondents from the surgery department of the Amsterdam UMC University Hospital, of whom 63.8% (n=30) were residents or PhD students and 36.2% (n=17) were surgeons. During one year of the combined EBS course and JCs, the EBS course was attended by 40.0% (n=12) of PhD students and was rated with a mean score of 7.6/10. JCs were attended by 86.6% (n=26) of residents or PhD students and received a mean score of 7.4/10. Reported strengths of the JCs were their easy accessibility and the acquisition of critical appraisal skills and scientific knowledge. A reported point of improvement was to focus more deeply on specific epidemiological topics per meeting. Of the surgeons, 64.7% (n=11) had supervised at least one JC and gave a mean score of 8.5/10. The main reasons to supervise JCs were the distribution of knowledge (45.5%), scientific discussion (36.3%), and contact with PhD students (18.1%).

Conclusion

Our EBS educational program, including JCs and EBS courses, was well appreciated by residents, PhD students, and staff. This format is advocated for other centers aiming to better implement EBS in surgical practice.

## Introduction

The principle of evidence-based medicine (EBM) was introduced in the early 1990s to provide clinicians with the ability to identify, retrieve, appraise, and integrate research evidence into medical decision-making [1]. Finding and critically appraising evidence-based clinical information is an essential skill for medical professionals. Hence, in medical education, EBM has gradually become a standard constituent of the curriculum [2,3]. EBM has also been structurally implemented in postgraduate medical and surgical education [4,5]. Nowadays, EBM is considered the established paradigm in medicine and surgery to provide the highest quality of care [6] but is not always embedded in postgraduate training and practice.

It has been argued, however, that in surgical specialties, as compared to other medical specialties, additional obstacles exist to the implementation and practice of EBM. Such obstacles range from the technical or ethical considerations involved in conducting randomized clinical trials in surgery to reluctance to change some of the long-established surgical practices [6,7]. On the other hand, technology and medical knowledge are changing rapidly, and the number of articles published each year is in the hundreds of thousands. It is hardly possible for any clinician to keep abreast of all the scientific literature pertinent to their specialty. In addition, the quality of the surgical literature is variable, so surgeons need to be selective in what they read. In order to do so, surgeons must have the skills to choose the best and most relevant articles and to appraise the articles critically to check whether the conclusions are valid and applicable to their own practices. Hence, education and training are essential to obtaining and maintaining these skills.

However, presenting this EBM paradigm in an integrated and relevant manner remains challenging [8], in particular regarding critical appraisal skills [9]. The use of a multifaceted approach that includes journal clubs, workshops, or online sessions may be helpful in increasing skills and confidence when teaching and practicing EBM [10,11]. 

A study in our institution, published in 2016, showed the merits of a two-day evidence-based surgery course (not including journal clubs) [5]. In this study, knowledge and skills regarding clinical epidemiology and critical appraisal of various study designs were investigated among surgical residents, showing clear improvements immediately after the course. Not long after this publication, the course was divided into four half-day modules within a year to enable more participants to join, as a two-day course hardly fitted in the residents' busy schedules. Also, monthly journal clubs were added, particularly to involve the PhD students as well, considering these critical appraisal exercises would improve their research and writing skills.

As a journal club is a well-established and popular method of post-graduate education in EBM, in particular critical appraisal, it may be a valuable addition to the evidence-based surgery course. Journal clubs are generally referred to as digital or face-to-face gatherings of interested individuals to discuss the medical literature and teach critical appraisal skills, and are one of the most frequently used traditional methods for teaching EBM [12,13]. Surveys in various surgical specialties, including general surgery, neurosurgery, and orthopedics, have all reported high ratings of the educational value of journal clubs by both residents and program directors [14-16]. Several ways of optimizing a journal club have been proposed [17], but the ideal formula is still unclear [10]. Therefore, an insight into health professionals’ experiences of journal clubs as a form of education is helpful.

Given the changes made in the way we offered the evidence-based surgery course and journal clubs, we felt the need to investigate in the present study how surgical PhD students, staff members, and residents perceive a modular evidence-based surgery course in combination with journal clubs and how they evaluate their personal process of acquiring new scientific knowledge and skills as an indispensable prerequisite of their lifelong learning. This could help improve future evidence-based surgery initiatives and might encourage other centers to adopt these practical teaching methods.

This article was previously presented as a meeting abstract at the 2022 International Joint Conference of Healthcare Professionals, Bandos, Maldives, on July 19, 2022.

## Materials and methods

Design and setting

This study was conducted in April 2022 as an anonymous digital survey, distributed among surgeons, PhD students, and residents of the surgery department of the Amsterdam University Medical Centers, located in the Academic Medical Center, Amsterdam, who had been able to attend the course and journal clubs during the past year. Available mailing lists of the staff members, PhD students, and residents were used to distribute the questionnaire. Participants who had attended more than one meeting were allowed to complete the questionnaire only once. The department offers gastrointestinal, vascular, hepato-pancreato-biliary, endocrine, and trauma surgery and has around 40 staff members, 30 first-year residents, and about 100 PhD students, although not all are involved in patient-oriented topics.

The local medical ethics review board waived the need for a full review of this study as it did not involve patients and did not hamper routine patient care.

Evidence-based surgery course

The evidence-based surgery course has been provided for over 20 years in our department and now consists of four modules: 1) observational studies in surgery; 2) diagnostic accuracy studies; 3) systematic reviews; and 4) weighing the benefits and harms of a surgical intervention, based on a randomized clinical trial, also to facilitate shared decision-making. Every quarter, one of these modules is presented, and the course is repeated every year. A surgeon and a clinical epidemiologist lead the sessions, which take about three hours. In these sessions, they present a general introduction about the particular study design, followed by an interactive discussion about the critical appraisal of the article, including the impact of the findings on clinical practice and how to share this evidence with patients.

To prepare for each module, participants receive an EBM manual with some background papers (e.g., about study types, critical appraisal, and outcome measures) and exercises (extracting data, performing a critical appraisal of study validity, interpreting the results, and assessing applicability). The course is voluntary for PhD students but mandatory for first-year residents in training; a small fee is required from both groups. Participants who have attended all four sessions receive a certificate that can be added to their portfolio for their PhD or surgical education.

Journal club

The journal clubs, which have been organized monthly for the last five years, are completely voluntary, free of charge, and take one hour each. They are led by one of the PhD students and are supervised by a staff surgeon and/or clinical epidemiologist. The paper selected for critical appraisal should have been recently published and was chosen by the PhD student in charge, in consultation with the supervising staff. The article should be timely and relevant for surgical practice and should address a particular methodological challenge, for example, the intention-to-treat principle, stepped-wedge cluster randomization, non-inferiority design, confounding, etc.

Participants receive the article and the validated critical appraisal checklist appropriate for the article’s study design (www.cochrane.nl/resources) by email, at least one week before the journal club, in order to be well prepared. During the journal club, the paper is discussed and critically appraised regarding its methodological validity, the clinical relevance of the results (i.e., beneficial and harmful effects), their precision, and the applicability of the findings to their own setting and patient population.

Survey

The custom-made, in part quantitative, digital survey addressed participation, satisfaction, and knowledge gained by the participants. It included seven general questions on previous EBM education, 14 specific course-oriented questions for the residents and PhD students, and seven questions for surgeons about supervising the journal clubs (see Appendix 1). The prototype of the questionnaire was scrutinized by the course teachers. The questionnaire was then sent out via email, and the initial non-respondents were reminded twice.

Data analysis

Baseline demographics were analyzed using basic statistics and were expressed as means or medians (with interquartile ranges) wherever appropriate, or as percentages for dichotomous variables. Statistical analysis was conducted using IBM Statistical Package for Social Sciences (SPSS) Statistics for Windows, Version 28.0. Armonk, NY: IBM Corp. Written feedback was appreciated qualitatively.

## Results

The questionnaire was completed by 28 surgical PhD students (i.e., 22% of all currently present PhD students), two junior residents, and 17 surgical staff members (i.e., 43% of the current number of staff members). Their characteristics are shown in Table 1, in which data from PhD students and residents were combined.

**Table 1:** Participant characteristics

	PhD students /Residents	Staff members
Number	30	17
Age (mean; range)	28.1 (23-31) years	46.8 (38-61) years
Male (%)	13/30 (43.3%)	12/17 (70.6%)
Clinical experience (median; interquartile range)	1 (1-2) years	16 (15-20) years
Research experience (median; interquartile range)	2 (1-3) years	20 (19-29) years
Obtained master's in Clinical Epidemiology (%)	2/30 (6.7%)	4/17 (23.5%)

Journal Clubs

Typically, eight to 15 participants attended each journal club. Some 87% of the participants attended at least one journal club, most of them three to 10 times. Residents and PhD students gave them an overall score of 7.4 out of 10. Topics discussed represented a range of relevant surgical issues: upper and lower gastrointestinal surgery, hepato-pancreato-biliary (HPB) surgery, and vascular surgery. Reported strengths of the journal club were its easy accessibility, the acquisition of critical appraisal skills (facilitated by using the checklists), and methodological knowledge. On the other hand, a desire was expressed for a deeper focus on a single methodological issue in each journal club. Participants preferred a face-to-face meeting to a digital one. They also suggested making the sessions mandatory to increase attendance.

Of the surgical staff members, 67.7% attended at least one journal club; most of them had supervised one to three meetings. They gave them an overall score of 8.5 out of 10. When asked about their drive to participate, 45.5% answered "to distribute knowledge", 36.6% mentioned "to facilitate a scientific discussion", and 36.6% stated their wish to have contact with PhD students. The strengths mentioned by the staff were the interaction and input in the journal clubs and the combination of clinical and research activities. The suggestions for improvement focused more on the clinical impact and the possibility of changing policies based on the evidence presented in the paper. Also, the journal club could be even more profitable by writing a letter to the editor of the journal in which the paper had been published. Besides, addressing a broader range of surgical topics was advocated.

Evidence-Based Surgery Course

Approximately 40% of all PhD students and residents attended at least one of the quarterly evidence-based surgery modules. They rated them an overall score of 7.6 out of 10. The main reasons for attending none or only a few modules were lack of time or having just started. The positive aspects reported were the well-structured conduct and a good focus on the most relevant methodological topics related to surgical research. This was seen as a valuable addition to the journal clubs. The discussions in small groups as part of the modules were appreciated. Those who had already obtained a master's degree in clinical epidemiology stated they had not gained much additional knowledge from the EBS course. Here also, the participants preferred face-to-face meetings over virtual meetings. As a suggestion for improvement, they proposed to create e-learnings to prepare for the session rather than a paper-based or digital manual.

## Discussion

This survey showed that the combination of a modular evidence-based surgery course with regular journal clubs was appreciated by surgical staff, PhD students, and residents. This was illustrated by the high attendance rate. The meetings were considered easily accessible, with interactive discussions and input. The evidence-based surgery course was also appreciated as it deepened methodological knowledge, although it did not go beyond the master's level, which is a limitation for those who have already obtained their master’s degree in clinical epidemiology. Also, not all steps of EBM were addressed in the course, as the vast majority of the participants were already proficient in formulating clinical questions (based on PICO (population, intervention, control, and outcomes)) and searching for evidence in the MEDLINE (Medical Literature Analysis and Retrieval System Online) and Cochrane databases. The merits of a journal club could be further enhanced by zooming in on a specific methodological or epidemiological issue and addressing the impact on patients in clinical practice, and by using the outcome of the critical appraisal as input for a letter to the editor.

So far, evidence in the literature has been controversial as to the impact of journal clubs on critical appraisal skills [10,18], and the understanding of biostatistics in (surgical) residents [19]. Others have shown results that are more positive in postgraduate medical students [20]. Providing a short checklist to interpret the validity, results, and applicability of each separate study design, as provided, for example, by the National Cochrane Center (www.cochrane.nl/resources), appeared to be helpful. More recently, open educational resources (OERs) have been proposed for residents to appreciate new evidence [21]. These medical educational facilities via the Internet may offer a more dynamic experience, allowing easier participation in discussions, even for junior residents, with a wider audience beyond the borders of their own hospital.

Implications for surgical practice

The journal clubs were an extension of our long-standing evidence-based surgery courses for PhD students [5]. Involving staff, PhD students, as well as residents in the journal clubs is needed to promote further integration of the EBM principle throughout the department. Critical appraisal of available evidence has become a standard part of the weekly and monthly research meetings and case discussions. In addition, the EBS course and a yearly EBM knowledge test have become obligatory courses in the education of surgical residents in training to become surgeons. Journal clubs are also a starting point for reviewing and updating local surgical protocols. Apart from EBM training and journal clubs, support from the management and a helpful education system has been shown to be essential prerequisites to improving skills, knowledge, and confidence in EBM [22].

Broadly speaking, to ensure that EBM principles and skills take root in clinical practice, journal clubs should be an integral part of both preclinical and postgraduate education [23], not only in medicine but also in nursing [24]. In addition, integrating shared decision-making into the EBM course can add a more person-centered care focus that would provide more clinical balance to the program. [25,26].

Study limitations

Although the journal clubs and EBS courses were welcomed by the participants, it would also be interesting to see what the direct impact is on knowledge, skills, and daily clinical practice. A previous study in our center on the effect of the EBS course on the participants’ knowledge and skills regarding epidemiological terms and calculations of outcome measures was found to increase significantly shortly after every course [5]. Metrics to assess EBM behavior have been reported, but few of these appreciate all steps of EBM [27]. In this study, we refrained from assessing the participants' knowledge or skills, which would have been much more complicated (possibly through an interrupted time series analysis) than in our previous study about the two-day course, as in the current situation there is no clear endpoint of the journal clubs.

This survey was conducted in an era where COVID initially necessitated digital meetings, which may have hampered interaction and discussion, but eventually, face-to-face meetings were allowed. Being able to compare those two phases led the participants to prefer face-to-face sessions to foster personal and scientific interaction.

The response rate of this survey was limited, especially among residents, but its denominator is debatable. Those who last attended the modules and journal clubs over one year ago were not invited in order to avoid recall bias. Others could not be invited as they had moved to another hospital. Moreover, clinicians and researchers are frequently invited to participate in surveys and questionnaires for many studies, which may have led to some "survey fatigue". However, responses were obtained from participants as well as non-participants. Hence, we believe the impact of possible selection bias on the results is small.

As this was a cross-sectional study, we cannot compare it to previous data to appreciate any improvement in the knowledge and application of EBM over time.

## Conclusions

The principles of evidence-based medicine including critical appraisal are essential to keep abreast of the new developments and available evidence in clinical practice in order to to ensure high-quality healthcare. Education in study methodology and practical skills through journal clubs seem synergetic for future clinicians in surgery. In the future, a regular checklist or test may be useful to assess the knowledge and progress of care professionals and researchers in surgery as to the application of EBM principles.

## Appendices

Appendix 1

Survey Items

General

- PhD-student, resident, staff (if so, redirected to staff-specific questions below)?

- Age

- Male/female

- Working experience (research and clinical)

- Previous training in clinical epidemiology

- Experience in writing any of the following study designs?

     o Systematic Review (+ meta-analysis)

     o Series/Cohort studies

     o Case reports

     o Qualitative research

     o Randomized clinical trials

Evidence-based surgery course

- Are you familiar with the EBS course?

     o Yes o No

- Are you, or have you been, participating in the EBS course?

     o Yes o No

- If no, why not?

     o On the waiting list

     o Didn’t know it existed

     o No desire to participate

     o No time to participate

     o Other reason being: 

- If yes, what is your main reason for participating?

     o Content

     o Contact with colleagues

     o Educational obligation

     o To obtain education points

     o Other reason being:

- If yes, how would you rate the EBS course on a scale from 0-10?

     o 0-10

          If 0-6: what must be improved?

          If 7-8: what can be improved?

          If 9-10: why so satisfied?

- If yes, would you recommend the EBS course to a colleague?

     o Yes o No

Journal clubs

- Are you familiar with the journal club meetings?

     o Yes o No

- Have you been participating in journal clubs?

     o No

     o 1-3 times

     o 4-10 times

     o More than 10 times

- If No or 1-3: what is the reason you participated up to 3 times?

     o Didn’t know it existed

     o I just started participating

     o No desire to participate

     o I am already experienced; has no added value for me

     o No time to participate

- If 4 or more: what is your main reason for participating?

     o Content

     o Contact with colleagues

     o Educational obligation

     o To obtain education points

- If participating, what do you learn in the Journal Clubs?

     o Study methodology

     o Statistics

     o Discussing the value of a paper

     o Structured appraisal of a paper

- How would you rate the Journal Clubs on a scale from 0-10?

     o 0-10

          If 0-6: what must be improved?

          If 7-8: what can be improved?

          If 9-10: why so satisfied?

- If participating, would you recommend the Journal Club to a colleague?

     o Yes o No

- Are there any topics you would like to be discussed (more)?

     o No o Yes: …

Staff

- Are you familiar with the journal club meetings?

     o Yes o No

- Did you ever supervise a journal club?

     o No

     o Yes, 1-3 times

     o Yes, 4-6 times

     o Yes, over 6 times

- If no, why not?

     o I still want to

     o Didn’t know it existed

     o No time

     o Don’t see added value

     o Other reason being: 

- If yes, why?

     o To share knowledge

     o Contact with PhD-students

     o Nice discussions

     o (Moral) obligation

     o Other reason being: 

- If yes, what was the main added value?

     o Study methodology

     o Statistics

     o Discussing the value of a paper

     o Structured appraisal of a paper

     o Other reason being: 

- What could we improve about the journal clubs?

- How would you rate the journal clubs on a scale from 0-10?

     o 0-10

          If 0-6: what must be improved?

          If 7-8: what can be improved?

          If 9-10: why so satisfied?

- Would you recommend the journal clubs to a colleague?

     o Yes o No
